# Predicting the Physiological Effect of Revascularization in Serially Diseased Coronary Arteries: Clinical Validation of a Novel CT Coronary Angiography–Based Technique

**DOI:** 10.1161/CIRCINTERVENTIONS.118.007577

**Published:** 2019-02-06

**Authors:** Bhavik N. Modi, Sethuraman Sankaran, Hyun Jin Kim, Howard Ellis, Campbell Rogers, Charles A. Taylor, Ronak Rajani, Divaka Perera

**Affiliations:** 1 NIHR Biomedical Research Centre and British Heart Foundation Centre of Excellence, School of Cardiovascular Medicine and Sciences, King’s College London (B.N.M., H.E., R.R., D.P.); 2 HeartFlow Inc, Redwood City, California (S.S., H.J.K., C.R., C.A.T.).

**Keywords:** contraindications, coronary angiography, coronary artery disease, hyperemia, percutaneous coronary intervention

## Abstract

**Background:**

Fractional flow reserve (FFR) is commonly used to assess the functional significance of coronary artery disease but is theoretically limited in evaluating individual stenoses in serially diseased vessels. We sought to characterize the accuracy of assessing individual stenoses in serial disease using invasive FFR pullback and the noninvasive equivalent, fractional flow reserve by computed tomography (FFR_CT_). We subsequently describe and test the accuracy of a novel noninvasive FFR_CT_-derived percutaneous coronary intervention (PCI) planning tool (FFR_CT-P_) in predicting the true significance of individual stenoses.

**Methods and Results:**

Patients with angiographic serial coronary artery disease scheduled for PCI were enrolled and underwent prospective coronary CT angiography with conventional FFR_CT_-derived post hoc for each vessel and stenosis (FFR_CT_). Before PCI, the invasive hyperemic pressure-wire pullback was performed to derive the apparent FFR contribution of each stenosis (FFR_pullback_). The true FFR attributable to individual lesions (FFR_true_) was then measured following PCI of one of the lesions. The predictive accuracy of FFR_pullback_, FFR_CT_, and the novel technique (FFR_CT-P_) was then assessed against FFR_true_. From the 24 patients undergoing the protocol, 19 vessels had post hoc FFR_CT_ and FFR_CT-P_ calculation. When assessing the distal effect of all lesions, FFR_CT_ correlated moderately well with invasive FFR (*R*=0.71; *P*<0.001). For lesion-specific assessment, there was significant underestimation of FFR_true_ using FFR_pullback_ (mean discrepancy, 0.06±0.05; *P*<0.001, representing a 42% error) and conventional trans-lesional FFR_CT_ (0.05±0.06; *P*<0.001, 37% error). Using FFR_CT-P_, stenosis underestimation was significantly reduced to a 7% error (0.01±0.05; *P*<0.001).

**Conclusions:**

FFR pullback and conventional FFR_CT_ significantly underestimate true stenosis contribution in serial coronary artery disease. A novel noninvasive FFR_CT_-based PCI planner tool more accurately predicts the true FFR contribution of each stenosis in serial coronary artery disease.

**WHAT IS KNOWN**

- Fractional flow reserve (FFR) is commonly used to assess the functional significance of coronary artery disease with noninvasive estimation using FFR by computed tomography (FFR_CT_) growing in popularity.
- Estimating the physiological significance of individual stenoses in the commonly encountered scenario of serial/diffuse coronary artery disease but is theoretically limited with FFR.

**WHAT THE STUDY ADDS**

- This study shows that conventional use of FFR and FFR_CT_ significantly underestimates true stenosis contribution in serial and diffuse coronary artery disease.
- This study describes a novel noninvasive FFR_CT_-based percutaneous coronary intervention planner tool (FFR_CT_).
- We show that FFR_CT_ more accurately predicts the true FFR contribution of each stenosis in serial coronary artery disease, compared with conventional FFR pullback and FFR_CT_ outputs.
- The findings of this study would support further large-scale studies examining the validity and applicability of this novel noninvasive percutaneous coronary intervention planning tool.

Coronary computed tomographic angiography (CTA) is now established as a noninvasive test for the detection of significant coronary artery disease (CAD).^1,2^ Owing to a relatively low positive predictive value and inability to determine functional significance,^2^ CTA has generally been used in patients at a low to intermediate risk with a view to ruling out significant CAD.^3^ When coronary atheroma is detected, there is growing evidence that the benefit of revascularization is only derived from targeting myocardial ischemia.^4^ Ischemia-guided revascularization has evolved to identifying vessels with functionally significant disease at the time of angiography using indices such as fractional flow reserve (FFR).^5–7^ In recent years it has become possible to estimate FFR from standard coronary CTA data sets with FFR by computed tomography (FFR_CT_). Based on computational fluid dynamics (CFD), FFR_CT_ has been shown to increase the positive predictive value of coronary CTA and provide clinicians with a noninvasive test to assess coronary anatomy and physiology.^8–10^

The studies validating both invasive FFR and FFR_CT_ have largely been in vessels with single epicardial stenoses.^9,11^ In reality, CAD is often diffuse in nature with serial stenoses across the length of a vessel. Assessment of the contribution of each stenosis is challenging because physiological interplay affects the FFR attributable to each stenosis.^12,13^ Coronary CTA enables detailed visualization of lesion geometry and a potential to evaluate the true pressure drop across a stenosis by modeling the hemodynamic interplay between serial stenoses.^13^ FFR_CT,_ therefore, offers the potential to predict the functional significance of individual stenoses within a serially/diffusely diseased vessel, and therefore, to predict the residual functional disease burden following PCI.

In this study, we describe a novel noninvasive FFR_CT_-derived PCI planning tool (FFR_CT-P_), which models physiological interplay between stenoses. This tool is based on a reduced order model informed by 3-dimensional simulations of blood flow to estimate post-PCI pressure loss. In this study we aimed to: (1) characterize the accuracy of estimating individual stenosis significance in serially diseased vessels using invasive FFR pullback and conventional FFR_CT_ outputs (usually presented as a color contour map of FFR change down a vessel) and (2) validate the novel noninvasive FFR_CT-P_ in a clinical cohort of patients with serial disease, with the aim of comparing the accuracy to estimations made from conventional FFR pullback and FFR_CT_.

## Methods

The data that support the findings of this study are available from the corresponding author on reasonable request.

### Study Population

All patients recruited to the study gave informed consent. Patients had presented with symptoms of stable angina and had ≥2 stenoses on an invasive coronary angiogram (>30% diameter stenosis by quantified coronary angiography, regardless of whether the stenosis represented a diffuse segment of disease or whether the stenosis was focal). Patients were only eligible if the segments of disease were separated by a normal segment of at least 10 mm, if it were felt feasible to treat each lesion independently by PCI. Exclusion criteria were age <18 years, pregnancy, estimated golmerular filtration rate <30, previous coronary artery bypass grafting, and contraindications to FFR assessment or CTA. Cardiac catheterization was performed within 4 weeks of the prior CTA scan. The study protocol was approved by the United Kingdom Health Research Authority and local research ethics committee (15/LO/2011).

### CT Protocol

All patients underwent coronary CT angiography using a 2×192-slice dual source CT scanner (Somatom Force, Siemens Medical Solutions, Forchheim), Germany. Eight hundred microgram of sublingual glyceryl trinitrate was administered to all patients along with intravenous metoprolol to achieve a heart rate of <65 bpm in sinus rhythm and <100 bpm in atrial fibrillation. A total of 85 mL intravenous contrast (Omnipaque, GE Healthcare, Princeton, NJ) was injected at 5.5 mL/s) by a power injector into the antecubital vein. Descending aorta contrast triggered, prospective ECG gated scanning with adaptive padding was then performed in a single breath-hold technique after a 10 to 12-second delay. The scanning parameters included a heart rate dependent pitch of 0.2 to 0.45 and a gantry rotation time of 250 ms. The tube voltage was selected semi-automatically, and automated exposure control was used for the tube current. Image slices were reconstructed using a medium sharp kernel (Bv40), using model-based iterative reconstruction strength level 3 (ADMIRE; Siemens Medical Solutions, Forchheim, Germany).

### FFR_CT_ Protocol

FFR_CT_ analysis was performed by HeartFlow, who were blinded to the invasive angiographic and physiological data. FFR_CT_ data were derived post hoc from the CTA data set using methodology that has been previously described.^9,14^ In summary, FFR_CT_ technology involves extraction of a patient-specific geometric model of the coronary arteries from CTA data. Subsequently, this is combined with population-derived physiological models and CFD techniques to solve the governing equations of blood flow velocity and pressure under simulated hyperemic conditions.^14^ FFR_CT_ physiological models are based on 3 core scientific principles: an allometric scaling law relating coronary flow to myocardial mass and vessel lumen volume, the principle of flow regulation of vessel size, and an assumption of the predictable reduction of microvascular resistance with maximal hyperemia.^15^

In this study, HeartFlow FFR_CT_ v2.7 was used. This software runs on the Amazon Web Services cloud and combines deep-learning artificial intelligence methods^16^ to create physiological models that incorporate vessel lumen volume and myocardial mass data.^15^ Normal HeartFlow FFR_CT_ outputs involve a color-coded chart of continuous FFR_CT_ values computed along each vessel. The apparent change in these discrete values across each stenosis within the serially diseased vessel was used to define the attributable FFR_CT_ for each stenosis within the serially diseased vessel, using conventional FFR_CT_ methods without the planning tool.

### Estimation of FFR_CT_ Post-Stenting

To enable the fast recalculation of FFR_CT_ for different planning configurations of PCI, an accelerated method for updating FFR_CT_ was used (based on a reduced order model derived from CFD simulations). Without compromising on the accuracy of FFR_CT_, this approach is able to update FFR_CT_ solutions in real-time in response to changes in lumen geometry. We ensured accuracy was not compromised by comparing the performance of FFR_CT-P_ against full conventional FFR_CT_ 3D simulations performed on the same models (N=80) and observed that the accuracy was statistically equivalent to that of FFR_CT_ (*P* value for equivalence based on 2 one-sided *t* test=0.01). Initially, idealized vessel lumen radii are calculated by first evaluating percent diameter stenosis and subsequently calculating the radius at which there would be no lumen narrowing (ie, to achieve a percent stenosis of zero). Following this, a patient-specific idealized model is constructed using the idealized lumen radii. Finally, a CFD-derived reduced order model is used to calculate the updated FFR_CT_ values.

This method involves CFD-derived reduced order model based on a flow-dependent resistance model for the epicardial arteries, *R*=*R*_in_+*R*_sl_*Q*, where *R*_in_ and *R*_sl_ are the intercept and slope of the resistance-flow relationship, *Q* is the flowrate and 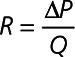 R= ΔPQis the resistance posed by the epicardial vessel to blood flow. Since these quantities also depend on the epicardial geometry, 2 different flowrates are used to calculate *R*_in_ and *R*_sl_. In each of the original and idealized geometries, 2 different boundary conditions, the first being hyperemia as described in the previous section and the second being a 40% lower resistance, are applied. If the flowrates achieved are *Q* and Q*, and the corresponding resistances calculated from CFD simulations are *R* and R* then:


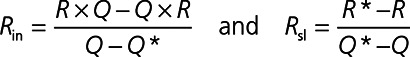


If the flow difference achieved, Q*−*Q*, is too low, then the intercept and slope are replaced with a 1D CFD model parameterized by lumen area (*A*), viscosity (*μ*), density (*ρ*), as


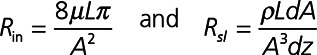


**Figure 1.**
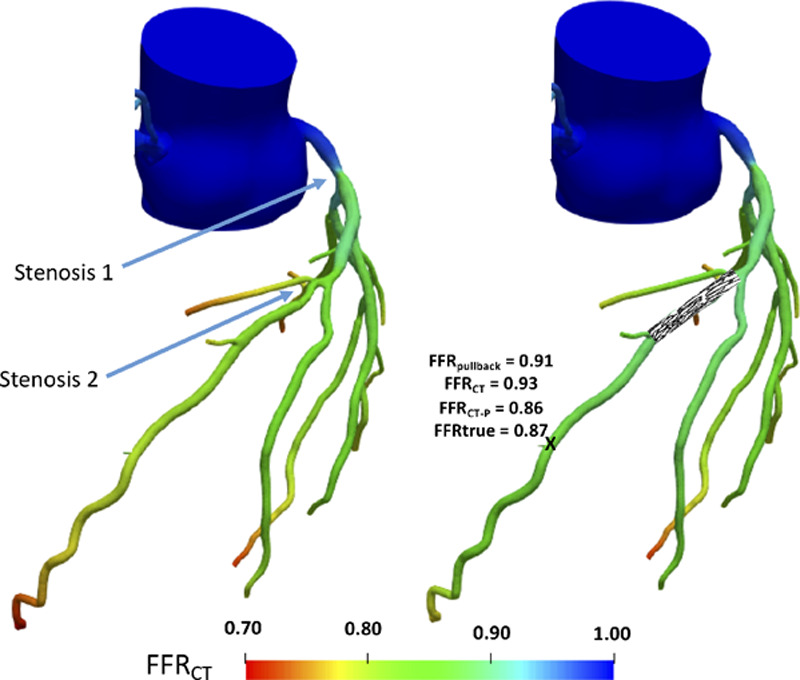
**Example case showing color contour map of fractional flow reserve (FFR) change in a serially diseased vessel with improvement in estimation of true stenosis significance with interactive planner tool. Left**, Example of conventional FFR by computed tomography (FFR_CT_) output for a serially diseased left anterior descending artery (serial stenoses labeled) before simulated percutaneous coronary intervention (PCI). The FFR_CT_ value between and distal to stenoses is suggested to be 0.93 and 0.80, respectively. **Right**, Results of true measured FFR at point X after PCI of stenosis 2, comparing favorably with FFR_CT_-derived PCI planning tool (FFR_CT-P_), following application of novel planner tool. Using conventional FFR_CT_ and FFR_pullback_ the underestimation would have been more significant (0.93 and 0.91, respectively).

These resistances are applied on the updated lumen geometry (where lumen radii in the stented region are replaced by their idealized values), to solve for flow rates, pressures, and FFR_CT_. For the purposes of this study, FFR_CT-P_ of each stenosis was derived following the retrospective application of these calculations to CTA images, blinded to invasive FFR values but with knowledge of stent size and position. Figure 1 illustrates the use of this FFR_CT_ planner tool in action.

### Cardiac Catheterization Protocol

Angiography was performed via the right radial artery using 6Fr catheters. Before catheterization, all patients received 300 mg of aspirin, 600 mg clopidogrel, and benzodiazepine sedation before local anesthetic was administered. Following arterial puncture, patients received 500 to 1000 μg isosorbide dinitrate into the radial artery before cardiac catheters were advanced and a further intracoronary bolus before the acquisition of standard diagnostic images.

### Invasive Pressure-Wire Data Protocol

Before insertion, the pressure-wire was zeroed outside of the body, against a fluid-filled guiding catheter pressure-transducer positioned at the level of the right atrium. After insertion, the pressure sensor was normalized to aortic pressure at the coronary ostium, with the guide disengaged where necessary. The pressure wire tip was delivered to the distal part of the serially-diseased vessel, beyond the distal stenosis (far enough to minimize the effect of post-stenotic turbulent flow), with wire position documented fluoroscopically. An IV adenosine infusion was administered at a dose 140 µg kg^−1^ min^−1^ via an antecubital vein. FFR was defined as the lowest Pd/Pa ratio averaged over 5 cardiac cycles following the onset of adenosine.^17^ Following documentation of FFR, the pressure-wire was pulled back to the guide catheter during continuous adenosine infusion at a constant speed (roughly 1 mm/s). This was done through the whole of the vessel, regardless of what the operator felt was a significant stenosis.

The apparent change in hyperemic pressure gradient across each stenosis following pressure-wire pullback within the serially diseased vessel was noted with the attributable FFR termed FFR_pullback_. PCI was then performed, with the choice of stenosis treated at the discretion of the operator using their conventional practice. Following re-calibration, pressure-wire pullback was then repeated (ensuring the stented segment no longer poses any pressure gradient). The subsequent true change in hyperemic pressure gradient across the remaining stenosis in isolation was noted with the stenosis-attributable FFR termed FFR_true_ (ie, FFR in the vessel following PCI of the accompanying serial stenosis). The difference between FFR_true_ and FFR_pullback_ was indicative of the degree of stenosis underestimation in serial CAD. The relationship between the extent of stenosis underestimation and total vessel FFR (the cumulative FFR in the vessel before PCI when both stenoses were present) was also assessed for each invasive and noninvasive index.

### Statistical Analysis

SPSS Version 23 (IBM Corp, Armonk, New York) and Prism Graphpad 5.0 (GraphPad Software Inc, CA) were used for all analyses. Normality was assessed using a visual assessment of histograms and Q-Q plots. Continuous data are expressed as mean±SD and compared using paired *t* tests. A 2-tailed test of significance was performed for all analyses with *P*<0.05 being considered statistically significant. Correlations were assessed using Pearson correlation coefficients (*R*). Bland-Altman analysis was used to compare the differences between estimated and true value across the range of measurements.

In addition, methods were compared in estimating the true post-PCI change in FFR across a stenosis. This was done by calculating the mean relative error (essentially the error as a proportion of the true FFR gradient across the lesion). This mean error is essentially calculated by evaluating the absolute difference between the true FFR gradient and the predicted FFR gradient (either using conventional FFR_CT_ or the novel FFR_CT-P_) and dividing this by the true FFR gradient across the stenosis in question.

## Results

**Figure 2.**
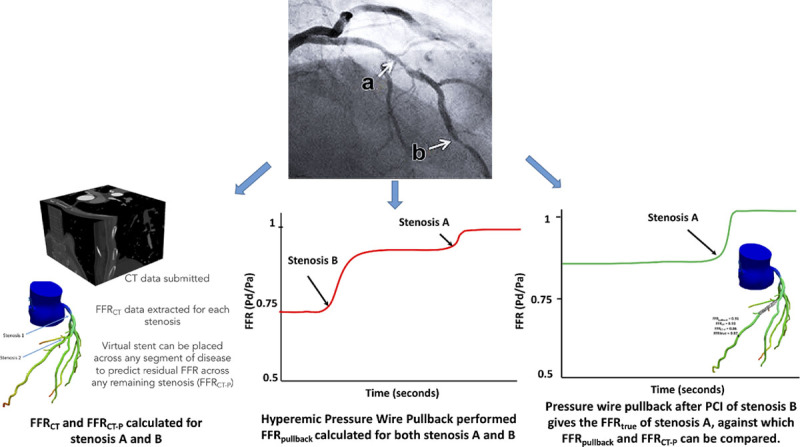
**Study flow diagram.** The Figure shows how fractional flow reserve by computed tomography (FFR_CT_)-derived percutaneous coronary intervention (PCI) planning tool (FFR_CT-P_) and FFR_pullback_ were calculated and subsequently compared with FFR_true_.

Twenty-seven patients had prospective CT coronary angiography that demonstrated serial CAD in 30 vessels. Of these, 5 patients (6 vessels) were excluded as satisfactory catheter laboratory data could not be obtained (reasons were: stenosis deemed too severe to make pressure-wire measurements, vessel tortuosity making it difficult to pass pressure-wire, and an adverse reaction to adenosine). Of the remaining 24 vessels, 19 (79%) had satisfactory computation of FFR_CT_ along the vessel (Figure 2 for study flow chart). Three patients were excluded because of proximal stented segments and 2 owing to CTA motion artifacts that precluded post hoc FFR_CT_ computation.

**Figure 3.**
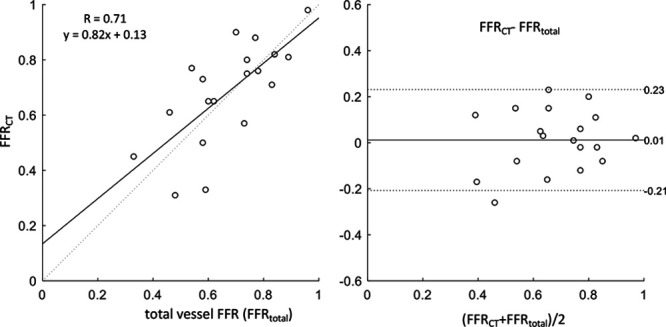
**Scatter and Bland-Altman plots comparing fractional flow reserve by computed tomography (FFR_CT_) against invasive FFR before percutaneous coronary intervention (PCI). Left**, Scatterplot comparing FFR_CT_ against invasively measured FFR before PCI. Solid lines represent the best linear fit to the data, and dotted lines represents the line of identity (x=y). **Right**, Bland-Altman plot comparing the differences between total vessel FFR_CT_ and total vessel FFR. Solid line represents the mean difference, and dotted lines correspond to the 95% CIs.

Patients included, in the final analysis, had a median age of 64.7 years (89% male). The Table summarizes the patient demographics and details of the serially diseased vessels. FFR of these vessels was 0.67±0.16. Total vessel FFR_CT_ of these vessels was 0.68±0.18. The Invasive total vessel FFR showed reasonable agreement with total vessel FFR_CT_, in line with previous studies^8,9^ (Pearsons *R*=0.71). Scatterplot and a Bland-Altman plot comparing invasive total vessel FFR with total vessel FFR_CT_ are shown in Figure 3.

**Table.**
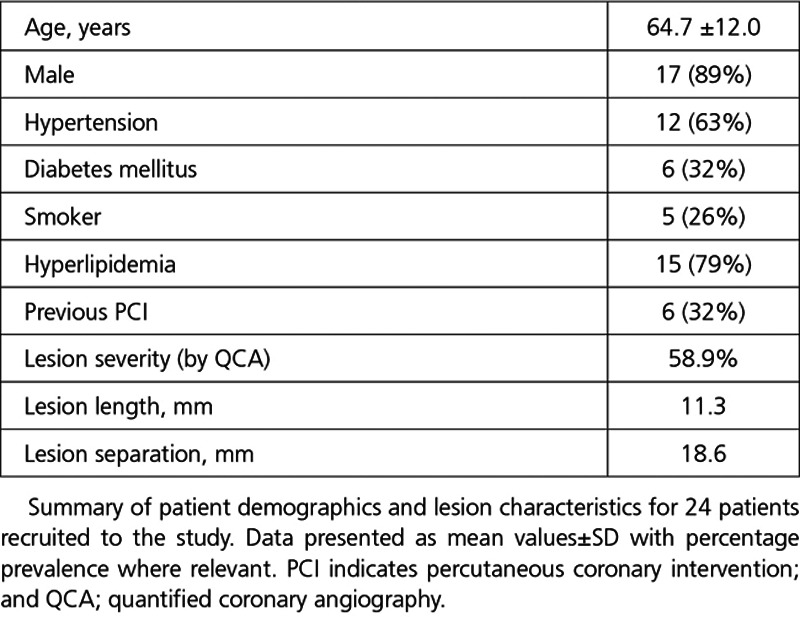
Summary of Patient Demographics and Lesion Characteristics

With FFR pullback, significant underestimation of stenosis contribution occurred, regardless of whether the proximal or distal lesion was considered, with a mean difference between FFR_pullback_ and FFR_true_ of 0.06±0.05 (*P*<0.001). This corresponded to a mean relative error of 42%. For conventional FFR_CT_ outputs along the course of vessels, similar significant underestimation of stenosis contribution occurred, with a mean difference between FFR_CT_ and FFR_true_ of 0.05±0.06 (*P*<0.001), giving a mean relative error of 37%.

### Estimating True Stenosis Significance With Interactive Planner Tool

Using the newly developed interactive FFR_CT_ PCI planning tool, we were able to estimate the FFR in the vessel FFR_CT-P_ following PCI of one of the 2 serial stenoses (example case shown in Figure 1). Optimal PCI was performed in 13 patients, with pressure wire pullback performed following PCI to ensure no residual lumen narrowing and pressure drop across the stented portion before assessing FFR_true_ (Figure 2 study flow chart).

**Figure 4.**
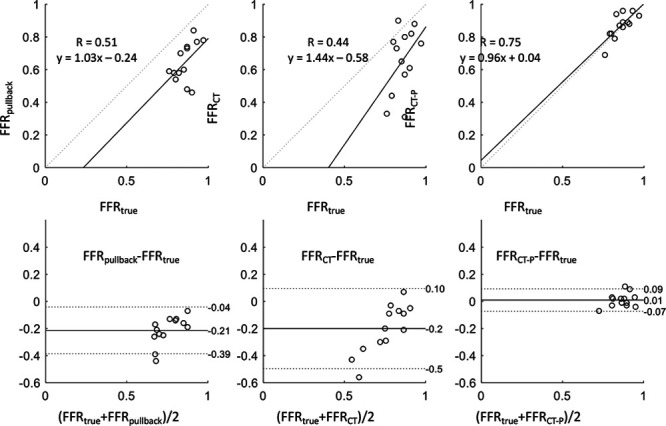
**Scatter and Bland-Altman plots demonstrating how planner tool improves estimation of FFR_true_. Top**, Scatterplots comparing the (left to right) FFR_pullback_, FFR by computed tomography (FFR_CT_), and FFR_CT_-derived percutaneous coronary intervention (PCI) planning tool (FFR_CT-P_) against true FFR. Solid lines represent the best linear fit to the data and dotted lines represents the line of identity (x=y). **Bottom**, Bland-Altman plot comparing the differences between FFR_true_ and FFR_pullback_, FFR_CT_, and FFR_CT-P_ (left to right). Solid line represents the mean difference, and dotted lines correspond to the 95% CIs. FFR indicates fractional flow reserve.

Use of FFR_CT-P_ resulted in a significant improvement in the correlation between the predicted FFR_CT_ and FFR_true_, following PCI of one of the 2 serial stenoses (R=0.44 with conventional FFR_CT_, improving significantly to *R*=0.75 with FFR_CT-P_; Figure 4). Whilst there was good direct correlation of per-stenosis FFR_CT-P_ to FFR_true_ (Pearson correlation coefficient 0.75, *P*=0.001; Figure 4), in a few cases there was significant over/under-estimation, particularly when the baseline total vessel FFR was <0.7). Figure 4 also illustrates the performance using FFR_pullback_. FFR_CT-P_ has a better correlation coefficient and a more linear fit to the data compared to FFR_CT_ or FFR_pullback_.

**Figure 5.**
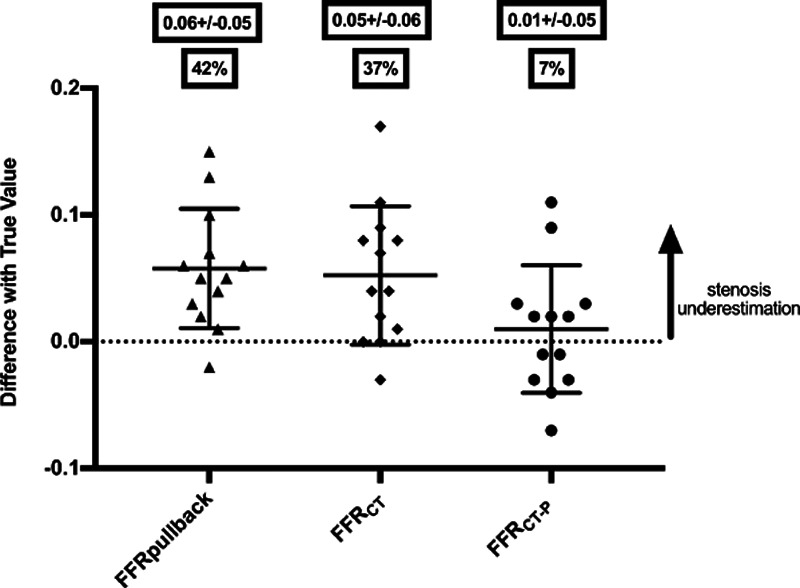
**Categorical scatter plot comparing errors in estimating true stenosis significance with different methods.** Categorical scatter plot comparing errors in estimating true stenosis significance (mean error±SD) with invasive FFR_pullback_ vs conventional FFR by computed tomography (FFR_CT_) vs results from FFR_CT_ with the percutaneous coronary intervention (PCI) planning tool (FFR_CT-P_). Also shown are percentage errors for each physiological tool as a proportion of the true FFR change, derived following isolation of a stenosis by PCI of an accompanying tandem stenosis. FFR indicates fractional flow reserve.

By applying the interactive planner tool on a geometry matching the stent location in the subsequent PCI, the absolute value of underestimation fell to 0.01±0.05, which corresponded to a mean proportional error of 7% (Figure 5). The equation performed equally well in the 2 cases where one of the 2 stenoses was diffuse (stenosis measured >30 mm in length by quantified coronary angiography).

## Discussion

In this study, we have shown that (1) using an unadjusted trans-lesional gradient from conventional FFR_CT_ outputs leads to significant underestimation of stenosis contribution in serially diseased vessels, similar to invasive FFR pullback methods and (2) applying a novel interactive PCI planning tool (FFR_CT-P_), results in a significant improvement in the accuracy of predicting the residual true FFR contribution of stenoses following treatment of accompanying serial stenoses. To our knowledge, this is the first description and validation of this novel noninvasive method to predict the hemodynamic outcome of a PCI procedure.

Serial and diffuse CAD is common, particularly within aging and diabetic populations, with estimates of 25% to 40% prevalence in all patients presenting for angiography.^18,19^ There are, however, several well-established limitations of physiological assessment of serial CAD. A simple explanation for this is that a stenosis provides resistance to blood flow, manifesting as a post-stenotic drop in pressure that is magnified in hyperemic conditions to give the true FFR when present in isolation. When another stenosis or diffusely-diseased segment, maximal hyperemia (and, therefore, measurement of the true FFR resulting from the stenoses) is not possible because of the additional resistance to flow, with the situation further complicated by flow turbulence when stenoses are particularly severe, close together and nonconcentric.^13,20^ The techniques previously described to overcome these limitations have been complex; requiring measurement of coronary wedge pressure^12^ or the presence of a disease-free daughter branch when serial disease involves the left main coronary artery.^21^ Although it has been suggested that resting indices (eg, iFR pullback) might result in smaller absolute errors than FFR in this setting,^22^ this is likely to be a reflection of the different operating range of resting indices, which are also prone to significant error.

At present, conventional FFR_CT_ outputs are presented as color-coded maps of coronary arteries showing how FFR_CT_ values change down the vessel. These color-coded gradients of FFR_CT_ are designed to mirror the changes in measured FFR one would expect with a hyperemic pressure-wire pullback within the catheter laboratory. In keeping with this, our results show that unadjusted trans-lesional gradients from these conventional FFR_CT_ outputs show a similarly significant degree of stenosis underestimation in serially diseased vessels, as is the case with invasive FFR pullback. Given that the physiological interplay largely depends on lesion geometry, which in turn can be well characterized by CTA techniques, a FFR_CT_-based technique would be expected to have greater potential at predicting the true physiological contribution of an individual stenosis, with the added appeal of being a noninvasive technique. Based on this, the FFR_CT-P_ has been developed to allow real-time recalculation of residual FFR after simulating PCI, with the operator able to choose simulated stent position and length.

Despite the large and significant reduction in mean error using the novel FFR_CT_ method (FFR_CT-P_), the variance of the error because of serial disease is still appreciable (SD ± 0.05), particularly when the cumulative burden of disease in the vessel is high (ie, total FFR low). These findings are consistent with previous meta-analyses of FFR_CT_ diagnostic performance that have shown reduced accuracy with more severe stenoses.^23^ This may be as a result of limited CT visualization of stenosis geometry when stenoses are severe but also variability in microvascular function which is difficult to predict from a noninvasive functional-anatomic test. In addition, the geometry of the untreated lesion can change post-PCI because of a higher operating pressure and can lead to unaccounted changes in FFR_CT_.^21^ There are several additional issues when using FFR_CT_ in planning intervention that may limit the utility of such a noninvasive tool: Firstly, there is a need to have an appropriate CT scan within a few weeks of the planned PCI. Secondly, not all prospectively acquired CT scans are of adequate quality for FFR_CT_ computation. Within our study, around 20% of cases could not have post hoc FFR_CT_ computation because of issues such as phase misalignment. This was a rate similar to the NXT trial where 13% of CT scans were unsuitable for analysis owing to inadequate image quality issues such as phase misalignment, stent-related artifacts, and blooming.^9^

Despite these factors, in patients that are able to have an adequate quality prospective CT coronary angiogram before planned PCI, FFR_CT-P_ still represents a significant improvement to current methods of physiologically assessing serial CAD. Furthermore, it has the potential to complement contemporary methods of planning PCI, particularly in patients with serial/diffuse CAD, without requiring the invasive positioning of a coronary guidewire. Further multi-center data will be needed to evaluate this tool and its impact of clinical decision-making.

### Limitations

1. The study is limited by its relatively small sample size and, therefore, the results are primarily hypothesis generating and merit further examination in a larger population derived from a multicenter setting.
2. In addition, FFR_CT_ has not been validated for patients with previous coronary artery bypass, previous stenting within the same vessel, or significantly abnormal left ventricular function, and therefore, by extension we excluded these patients, and we cannot extrapolate the results from the FFR_CT_ PCI planning tool to those patient groups.

## Conclusions

FFR_CT_ provides a comparable estimation of FFR values obtained from routine invasive pressure-wire pullback along a vessel and is prone to a similar degree of physiological underestimation in serial CAD. In this study, we provide validation for a novel noninvasive FFR_CT-P_ and show it to significantly reduce the error of contemporary methods in estimating true stenosis contribution within serially diseased vessels following PCI of an accompanying stenosis.

## Acknowledgments

David Lesage (employee at HeartFlow Inc) helped generate the FFR_CT_ patient-specific idealized lumen radii models. We acknowledge the ongoing support of the British Heart Foundation in supporting cardiovascular research in our institution and the United Kingdom as a whole.

## Sources of Funding

B. Modi is funded by a British Heart Foundation Clinical Research Training Fellowship (FS/15/78/31678) and D. Perera is supported by the United Kingdom National Institutes for Health Research via the Biomedical Research Centre award to King’s College London.

## Disclosures

Heart Flow Inc have borne the costs of post-processing CTA data. S. Sankaran, H.J. Kim, C. Rogers, and C.A. Taylor are Heart Flow Inc employees.
